# Systemic Insulin Resistance and Metabolic Perturbations in Chow Fed Inducible Nitric Oxide Synthase Knockout Male Mice: Partial Reversal by Nitrite Supplementation

**DOI:** 10.3390/antiox9080736

**Published:** 2020-08-12

**Authors:** Hobby Aggarwal, Priya Pathak, Pragati Singh, Jiaur R. Gayen, Kumaravelu Jagavelu, Madhu Dikshit

**Affiliations:** 1 Pharmacology Division, CSIR-Central Drug Research Institute, Lucknow 226031, India; hobby.agg@gmail.com (H.A.); priyapathak87@gmail.com (P.P.); kumaraveluj@cdri.res.in (K.J.); 2 Pharmaceutics & Pharmacokinetics Division, CSIR-Central Drug Research Institute, Lucknow 226031, India; pragati.bhu@gmail.com (P.S.); jr.gayen@cdri.res.in (J.R.G.); 3 Translational Health Science and Technology Institute, NCR Biotech Science Cluster, 3rd Milestone, Faridabad-Gurgaon Expressway, Faridabad, Haryana 121001, India

**Keywords:** iNOS^−/−^, nitrite, nitric oxide, insulin resistance, dyslipidemia, liver, adipose tissue, metabolism

## Abstract

iNOS, an important mediator of inflammation, has emerged as an important metabolic regulator. There are conflicting observations on the incidence of insulin resistance (IR) due to hyperglycemia/dyslipidemia in iNOS^−/−^ mice. There are reports that high fat diet (HFD) fed mice exhibited no change, protection, or enhanced susceptibility to IR. Similar observations were also reported for low fat diet (LFD) fed KO mice. In the present study chow fed iNOS^−/−^ mice were examined for the incidence of IR, and metabolic perturbations, and also for the effect of sodium nitrite supplementation (50 mg/L). In IR-iNOS^−/−^ mice, we observed significantly higher body weight, BMI, adiposity, blood glucose, HOMA-IR, serum/tissue lipids, glucose intolerance, enhanced gluconeogenesis, and disrupted insulin signaling. Expression of genes involved in hepatic and adipose tissue lipid uptake, synthesis, oxidation, and gluconeogenesis was upregulated with concomitant downregulation of genes for hepatic lipid excretion. Nitrite supplementation restored NO levels, significantly improved systemic IR, glucose tolerance, and also reduced lipid accumulation by rescuing hepatic insulin sensitivity, glucose, and lipid homeostasis. Obesity, gluconeogenesis, and adipose tissue insulin signaling were only partially reversed in nitrite supplemented iNOS^−/−^ mice. Our results thus demonstrate that nitrite supplementation to iNOS^−/−^ mice improves insulin sensitivity and metabolic homeostasis, thus further highlighting the metabolic role of iNOS.

## 1. Introduction

Nitric oxide (NO), a pleiotropic gaseous signaling molecule, plays an important role in the cardiovascular and metabolic regulations. NO synthesis is catalyzed by Ca^2+^ dependent constitutive NOS (eNOS and nNOS), and Ca^2+^ independent inducible iNOS [1]. Importance of iNOS was primarily studied in infectious diseases, and also in inflammatory conditions [2,3,4,5]. Subsequent studies, however, demonstrated constitutive presence of iNOS in various insulin responsive tissues such as liver [6], adipose tissue [7], skeletal muscle [8], and non-responsive tissues/cells like ileum [9], colon [10], and neutrophils [11]. Type 2 diabetes is a complex disease and is exceedingly heterogeneous in its manifestations with various subtypes necessitating detailed understanding of the mechanisms involved in its pathophysiology for its better management [12]. Insulin resistance (IR) is the key feature of obese diabetics due to altered glucose and lipid homeostasis [13]. Interestingly, genetic polymorphism at the iNOS gene (14-repeat allele) is linked to increased iNOS activity which confers selective advantage to diabetic individuals. This points towards the protective role of constitutive iNOS in preventing or delaying the pathological alterations in diabetes [14,15]. Studies from our group and others on the effect of iNOS derived NO on endothelium functionality have shown that acetylcholine [16] and insulin [17] mediated vasorelaxation was significantly preserved in obese iNOS^−/−^ mice, which was independent of IR, dyslipidemia or hyperglycemia, blood pressure, or oxidative stress. Reduction in NO bioavailability contributes to the pathogenesis of hyperlipidemia, endothelial dysfunction, atherosclerosis, hypertension, diabetes, and obesity [18,19,20].

Metabolic perturbations and altered insulin sensitivity have been commonly observed in eNOS, nNOS, and triple NOS KO mice [21,22,23]. Most of the studies on iNOS^−/−^ mice have demonstrated its definitive role in inflammatory [3,5] and infectious conditions [4,24] whereas inconsistent results were reported for its metabolic role [16,25,26,27,28,29,30,31,32,33]. HFD fed iNOS^−/−^ mice were protected from infiltration of pro-inflammatory macrophages and adipose tissue fibrosis [28]. Likewise, iNOS inhibition reversed hepatic IR and hyperglycemia in obese diabetic mice [34]. These studies suggest that iNOS/NO play an important role in the initiation of IR. Moreover, iNOS^−/−^ mice showed attenuated fructose induced-hepatic steatosis [26], dyslipidemia, IR, and nitrosative stress [27]. Fat deposition in the rat liver and circulatory lipids were, however, increased following iNOS inhibition [35,36]. Likewise, iNOS^−/−^ mice on long term HFD feeding exhibited increased adiposity [25,28,29], and fasting hyperglycemia despite being protected against systemic IR [25]. Our previous studies on LFD or HFD diet fed iNOS^−/−^ mice found them to be IR and also exhibiting perturbed metabolic homeostasis [16,32,37]. Additionally, iNOS^−/−^ mice fed on HFD or LFD displayed significant weight gain, higher fat mass, and dyslipidemia with reduced lean mass [33]. Even chow fed iNOS^−/−^ mice had higher fat mass [30] and circulating triglycerides levels [31]. Above contradictory observations may be due to differing dietary composition, regimens, and also the selection of control groups to interpret the results. In fact, dietary composition (high fat or sugar) can be the important reason of exaggerated inflammation and altered homeostasis.

Studies, both from our group and others, have shown that iNOS^−/−^ mice have decreased NO availability [32,38,39]. Nitrite, a precursor of NO reservoir, is abundant in green leafy vegetables and is also presumably protective against diabetes and cardiovascular diseases [21,40]. As iNOS^−/−^ mice exhibited reductions in the total nitrite content, it is hypothesized that nitrite supplementation in drinking water might compensate for the reduced NO availability. The present study thus investigates the incidence of IR in chow fed iNOS^−/−^ mice, and also the effect of nitrite supplementation on the rescue of systemic, hepatic, and adipose tissue insulin sensitivity. In the present study, the focus was on liver and adipose tissue as they are involved in the regulation of whole-body energy homeostasis and form a highly orchestrated metabolic circuit involving nutrient uptake, processing, transport, and storage. These tissues are shown to be important in the initiation of IR, while association of skeletal muscle has been highlighted in the later stage of disease [41].

## 2. Research Design and Methods

### 2.1. Animal Studies

Twelve weeks old, age matched male wild type(WT) and iNOS^−/−^ (Jackson Laboratory, Bar Harbor, ME, USA; 002609) mice on C57BL/6J background were bred and maintained in IVC cages (Tecniplast, Buguggiate, VA, Italy) at 24  ±  2  °C. All procedures were approved by Institutional Animal Ethics Committee of CSIR-CDRI (IAEC/2014/43) in accordance with CPCSEA guidelines. Mice (WT and iNOS^−/−^) were kept on chow diet (1320, Altromin, Lage, North Rhine-Westphalia, Germany) and glucose tolerance test was performed. WT and iNOS^−/−^ mice then received regular or sodium nitrite (50 mg/L, NaNO_2_) supplemented water ad libitum for 5 weeks as was reported earlier [42,43]. Body weight and length was measured and BMI calculated [44] at the end of 5 weeks of nitrite supplementation.

### 2.2. Tolerance Tests

Mice fasted for 6 h were administered 2 g/kg d-Glucose, 2 g/kg sodium pyruvate, or 0.6 IU/kg insulin (Human insulin R, Eli Lilly, Indianapolis, IN, USA) by intraperitoneal (i.p.) route for performing glucose (GTT), pyruvate (PTT), or insulin tolerance test (ITT). Blood glucose was monitored using a glucometer (Roche Diagnostics, Mumbai, Maharashtra, India) at 0, 15, 30, 60, and 120 min after administration of glucose, pyruvate, or insulin and area under the curve (AUC) was calculated as described previously [32].

### 2.3. Body Composition Analysis

Body composition (fat and lean mass) was analyzed by echo MRI (E26-226-RM Echo MRI LLC, Houston, TX, USA) in conscious live mice by applying radio frequency pulses at a distinct static magnetic field [32].

### 2.4. Indirect Calorimetry

Conscious, unrestrained mice were individually placed in the Oxymax CLAMS (Columbus Instruments, Columbus, OH, USA) with free access to food and water for metabolic measurements [45]. After pre-calibration of system and animal acclimatization, oxygen consumption (VO_2_, mL/kg/h), carbon dioxide produced (VCO_2_, mL/kg/h) along with food and water intake, RER (VCO_2_/VO_2_, m/kg/min), metabolic rates (BMR and RMR) and energy expenditure (kcal/h; heat production) were determined over a 3 day period.

### 2.5. Serum Biochemistry

Retro-orbital blood was collected from 6 h fasted mice. Estimation of lipids like total cholesterol (TC), triglycerides (TG), low and high density lipoproteins (LDL and HDL), and non-esterified fatty acids (NEFA) were performed in the serum using kits (Randox, Crumlin, Co. Antrim, UK, [16]). Insulin was measured using a kit from Crystal Chem, Elk Grove Village, IL, USA.

### 2.6. Total Nitrite Estimation

The animals were sacrificed to retrieve the tissues (liver, epididymal white adipose tissues, and hind limb skeletal muscle). Total nitrite (nitrate and nitrite) was estimated in serum (100 μL) and tissues (liver, eWAT, and skeletal muscle, 50 mg) using Griess reagent by reducing nitrate to nitrite using pre-activated cadmium pellets followed by deproteinization in tissue homogenates with 3% trichloroacetic acid [46].

### 2.7. Tissue Biochemistry

Liver tissue (50 mg) was processed as described previously for the estimation of hepatic total cholesterol [47], triglycerides [48], and free fatty acids [49] using Randox (Crumlin, Co. Antrim, UK) kit.

### 2.8. Hematoxylin and Eosin (HE) Staining

Formalin fixed and, paraffin embedded adipose tissue (eWAT) was sectioned into 5 μm thin serial slices and HE stained for morphological examination [45]. Adipocytes area was calculated using Adiposoft plugin in Fiji software for Windows 64bit (NIH, Bethesda, MA, USA).

### 2.9. Oil Red O Staining 

Formalin fixed, tissue freezing medium (Leica Biosystems, Ernst-Leitz-Strasse, Wetzlar, Germany) embedded frozen liver tissues were sectioned in 10 μm thin slices, stained with Oil Red O and counterstained with hematoxylin to visualize the lipid accumulation using Leica QWin version 3.5.1 software [47].

### 2.10. Western Blot Analysis and Real Time PCR

Insulin was administered at a dose of 0.6 IU/kg i.p. and animals were culled after 30 min to collect organs (liver and eWAT) for Western blot studies along with unstimulated controls in both WT and iNOS^−/−^ mice with or without nitrite supplementation. Liver and adipose tissue protein extracts were subjected to SDS-PAGE, transferred to PVDF membrane, and probed with primary antibodies against Akt or p-Akt^Ser473^ (details listed in Table S1) and visualized with chemiluminescence of horse radish peroxidase-linked secondary anti-rabbit or anti-sheep IgGs using ECL detection solution and normalized with β-actin. Quantitative densitometry was performed using Image J software for Windows 64bit (NIH, Bethesda, MA, USA). Real time PCR was performed as described previously [46] with primers listed in Table S2 and normalized with 18S rRNA.

### 2.11. Statistical Analysis

Data is presented as mean ± SEM. Independent unpaired Student’s *t* test was used for comparisons as appropriate using GraphPad Prism 7 software. Differences at *p* < 0.05 were considered statistically significant.

### 2.12. Data Availability

All data supporting the findings of this study are available from the corresponding author on reasonable request.

## 3. Results

### 3.1. Gross Parameters, Systemic Insulin, Glucose, Pyruvate Tolerance, and Circulating Lipids

**Figure 1:**
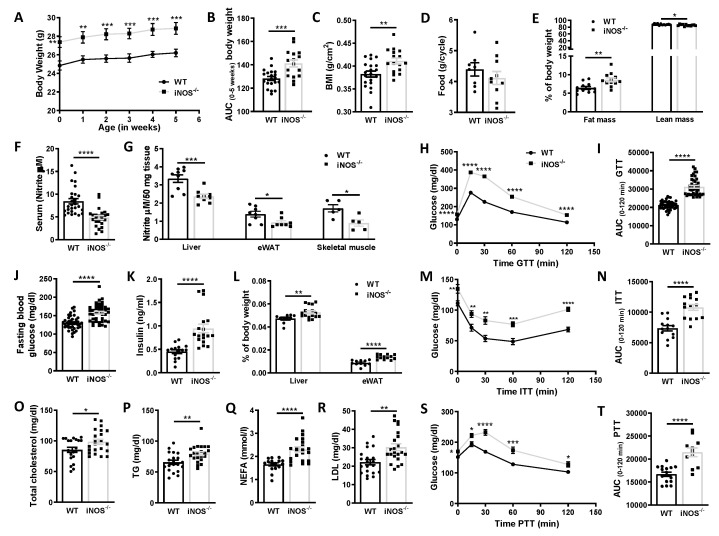
Gross parameters, systemic glucose tolerance, insulin sensitivity, gluconeogenesis, and circulating lipids in chow fed wild type (WT) and iNOS^−/−^ mice. (**A**) Body weight from the initiation (0 week) to study termination (5 weeks) (WT: *n* = 20, iNOS^−/−^: *n* = 16). (**B**) Area under the curve (AUC) calculated from the gradual change in the body weight of WT and iNOS^−/−^ (WT: *n* = 20, iNOS^−/−^: *n* = 16) mice. (**C**) Body mass index (BMI) (WT: *n* = 20, iNOS^−/−^: *n* = 16), (**D**) food consumption (WT: *n* = 8, iNOS^−/−^: *n* = 10), (**E**) whole body fat mass and lean mass (%) (*n* = 12), (**F**) total nitrite levels in serum (*n* = 24), (**G**) total nitrite levels in insulin sensitive tissues—liver (*n* = 9), white adipose tissue (*n* = 8), and skeletal muscle (*n* = 5). (**H**) Intraperitoneal glucose tolerance test (GTT) and (**I**) area under the curve (AUC) calculated from IPGTT data (*n* = 40). (**J**) Fasting blood glucose levels (*n* = 40), (**K**) fasting serum insulin levels (WT: *n* = 16, iNOS^−/−^: *n* = 18), (**L**) relative liver weight (WT: *n* = 10, iNOS^−/−^: *n* = 16), and epididymal white adipose tissue weight (eWAT) (WT: *n* = 11, iNOS^−/−^: *n* = 12). (**M**) Intraperitoneal insulin tolerance test (ITT) and (**N**) AUC calculated from ITT (WT: *n* = 12, iNOS^−/−^: *n* = 10). Serum lipid levels after 6 h fasting (WT: *n* = 16, iNOS^−/−^: *n* = 22). (**O**) Total cholesterol (TC), (**P**) triglycerides (TG), (**Q**) non-esterified free fatty acids (NEFA), (**R**) low density lipoprotein (LDL). (**S**) Intraperitoneal pyruvate tolerance test (PTT) and (**T**) AUC calculated from PTT (WT: *n* = 12, iNOS^−/−^: *n* = 10) in chow fed WT and iNOS^−/−^ mice. Data are represented as mean ± SEM. Black circles: WT, black squares: iNOS^−/−^ mice. * *p* < 0.05, ** *p* < 0.01, *** *p* < 0.001, **** *p* < 0.0001 vs. WT.

Chow fed iNOS^−/−^ mice at almost similar levels of food consumption weighed more, had higher BMI, body length, and fat mass while lean mass (Figure 1A–E and Figure S1G), VCO_2_/heat production, and metabolic rates (BMR and RMR, Figure S1A–C) were less as compared to WT mice. In iNOS^−/−^ mice, total nitrite contents in the serum, liver, adipose tissue, and skeletal muscle were significantly less (Figure 1F,G) along with decreased eNOS expression but with enhanced nNOS in the liver and adipose tissue (Figure S1D–F). Additionally, iNOS^−/−^ mice were glucose intolerant and also had higher circulating glucose levels (Figure 1J) as evident by the persisting increase in circulating glucose levels even 2 h after its administration (Figure 1H,I). iNOS^−/−^ mice also displayed systemic insulin resistance as evident by ITT, PTT (Figure 1M,N,S,T), and increased circulating insulin levels (Figure 1K), HOMA-IR, decreased QUICKI, and unchanged HOMA-B (Figure S1H–J). The relative liver and adipose tissue weights were higher in iNOS^−/−^ mice as compared to WT (Figure 1L). Circulating total cholesterol, triglycerides, NEFA, and LDL were significantly more in iNOS^−/−^ mice while HDL levels were comparable to WT mice (Figure 1O–R and Figure S1K).

### 3.2. Status of Metabolic Homeostasis in WT and iNOS^−/−^ Mice

#### 3.2.1. Metabolic Homeostasis in the Liver Tissue

**Figure 2:**
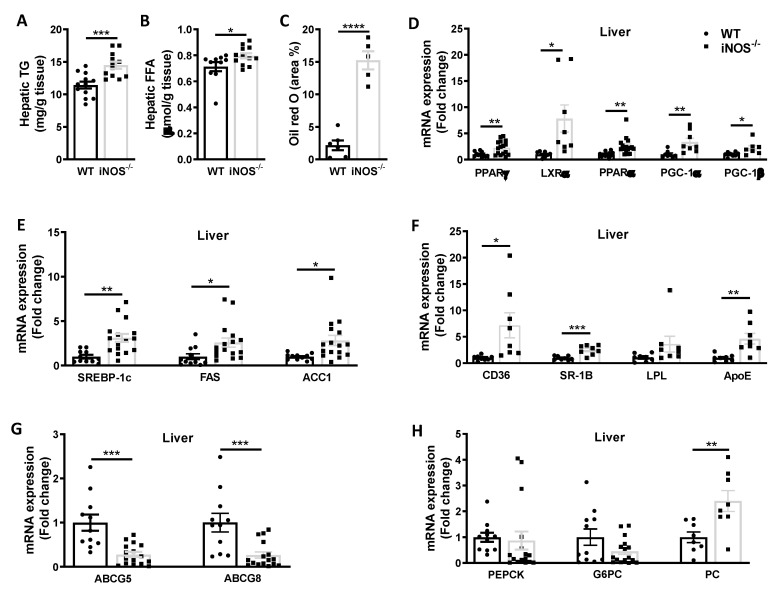
Metabolic homeostasis in liver of chow fed WT and iNOS^−/−^ mice. Lipid accumulation in liver. (**A**) Hepatic triglycerides (*n* = 12), (**B**) hepatic free fatty acids (WT: *n* = 10, iNOS^−/−^, *n* = 12) and (**C**) hepatic Oil red O staining (WT: *n* = 6, iNOS^−/−^, *n* = 5). (**D**) qPCR expressions of transcriptional regulators involved in lipid synthesis: PPARy (WT: *n* = 11, iNOS^−/−^, *n* = 16) and LXRα (*n* = 8) and genes involved in fatty acids oxidation: PPARα (*n* = 11–16), PGC-1α, and PGC-1β (*n* = 8). (**E**) qPCR expression of genes involved in lipid synthesis (WT: *n* = 11, iNOS^−/−^, *n* = 16): SREBP-1c, FAS, and ACC1. (**F**) qPCR expression of genes involved in lipid uptake (*n* = 8): CD36, SR-B, ApoE, and LPL. (**G**) qPCR expression of genes involved in lipid efflux (WT: *n* = 11, iNOS^−/−^, *n* = 16): ABCG5 and ABCG8. (**H**) qPCR expression of genes involved in gluconeogenesis (WT: *n* = 11, iNOS^−/−^, *n* = 16): PEPCK, G6PC, and PC (*n* = 8) in chow fed WT and iNOS^−/−^ mice. Data are represented as mean ± SEM. Black circles: WT, black squares: iNOS^−/−^ mice. * *p* < 0.05, ** *p* < 0.01, *** *p* < 0.001, **** *p* < 0.0001 vs. WT.

iNOS^−/−^ mice had higher lipid accumulation in liver as evident by significant increase in hepatic triglycerides, free fatty acids levels (Figure 2A,B), and Oil red O stained area (Figure 2C). qPCR analysis of transcriptional regulators of lipid synthesis and β-oxidation of fatty acids revealed significantly enhanced expression of PPARγ and LXRα, and PPARα, PGC-1α, and PGC-1β (Figure 2D). Similarly, the expression of genes involved in the triglyceride synthesis including SREBP-1c, FAS, and ACC1 was also more in KO mice as compared to WT mice (Figure 2E). Expressions of fatty acid uptake genes CD36, SR-1B, and ApoE were significantly enhanced in the liver of iNOS^−/−^ mice as compared to WT (Figure 2F). On the other hand, expression of lipid efflux genes, ABCG5 and ABCG8, was however significantly less in the liver of iNOS^−/−^ mice (Figure 2G). Moreover, enhanced gluconeogenesis in iNOS^−/−^ mice correlated with increase in the expression of PC while expression of G6PC, PEPCK, and transcriptional regulator FOXOA1 (data not shown) was not altered in the liver (Figure 2H).

#### 3.2.2. Metabolic Homeostasis in the Adipose Tissue

**Figure 3:**
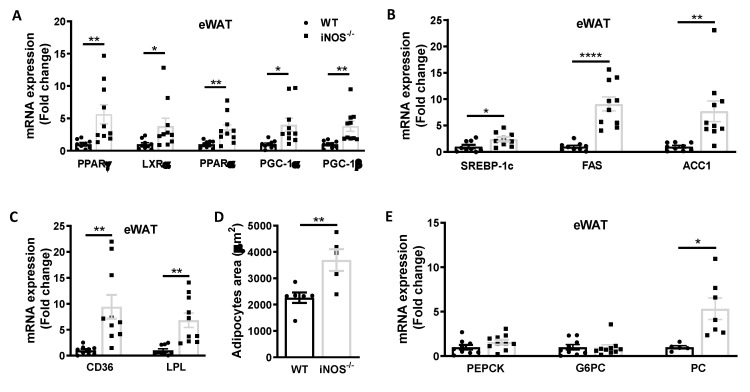
Metabolic homeostasis in adipose tissue of chow fed WT and iNOS^−/−^ mice. (**A**) qPCR expressions of transcriptional regulators involved in lipid synthesis (WT: *n* = 9, iNOS^−/−^: *n* = 10): PPARy and LXRα and genes involved in fatty acids oxidation: PPARα, PGC-1α, and PGC-1β. (**B**) qPCR expression of genes involved in lipid synthesis (WT: *n* = 9, iNOS^−/−^: *n* = 10): SREBP-1c, FAS, and ACC1. (**C**) qPCR expression of genes involved in lipid uptake (WT: *n* = 9, iNOS^−/−^: *n* = 10): CD36 and LPL. (**D**) Mean adipocyte area (WT: *n* = 6, iNOS^−/−^: *n* = 5). (**E**) qPCR expression of genes involved in gluconeogenesis (WT: *n* = 9, iNOS^−/−^: *n* = 10): PEPCK, G6PC, and PC (WT: *n* = 5, iNOS^−/−^: *n* = 7) in chow fed WT and iNOS^−/−^ mice. Data are represented as mean ± SEM. Black circles: WT, black squares: iNOS^−/−^ mice. * *p* < 0.05, ** *p* < 0.01, **** *p* < 0.0001 vs. WT.

Enhanced expressions of PPARγ, LXRα, PPARα, PGC-1α, and PGC-1β in adipose tissue suggest increase in lipogenesis and fatty acids oxidation in iNOS^−/−^ mice in comparison to WT (Figure 3A). Augmentation in the expression of lipogenic genes including SREBP-1c, FAS, and ACC1 was also evident in eWAT of KO mice (Figure 3B). Further, the fatty acid uptake gene-CD36 and lipolysis gene-LPL were also significantly upregulated in the adipose tissue of iNOS^−/−^ mice as compared to WT with increased adipocyte area (Figure 3C,D). Increase in PC (Figure 3E) with no change in the expression of PEPCK and G6PC (Figure 3E) was observed in the adipose tissue of iNOS^−/−^ mice.

### 3.3. Alterations in the Gross Parameters, Glucose Tolerance, Insulin Sensitivity, Gluconeogenesis, and Circulating Lipids after Nitrite Supplementation in Chow Fed iNOS^−/−^ Mice

iNOS^−/−^ mice displayed complete reversal in the nitrite content in serum, liver, skeletal muscle, and also adipose tissue upon nitrite supplementation (Figure S2A,B). At similar food/water consumption, and physical activity (data not shown), nitrite supplemented iNOS^−/−^ mice exhibited significant reduction in the gross parameters such as BMI, body weight, and fat mass but increased lean mass (Figure S2E–G). VCO_2_, heat production, BMR, and RMR along with body length remained unaltered in the nitrite supplemented group (Figure S3A–C,G). Nitrite supplemented iNOS^−/−^ animals showed decreased adipose tissue and liver weight (Figure S2J) whereas eNOS mRNA and protein expressions in the liver and adipose tissue were increased (Figure S3D,E) with no change in nNOS (Figure S3F) or iNOS expression (data not shown). Nitrite treatment significantly and partially improved systemic glucose intolerance and blood glucose levels in iNOS^−/−^ mice (Figure S2C,D,K). Nitrite supplementation also significantly improved the insulin sensitivity (Figure S2H,I) and restored insulin levels (Figure S2L), HOMA-IR, HOMA-B, and improved QUICKI (Figure S3H–J) in iNOS^−/−^ mice. Systemic gluconeogenesis was marginally reduced (*p* < 0.05) after nitrite supplementation in iNOS^−/−^ mice (Figure S2M,N). Nitrite supplementation also normalized serum triglycerides and NEFA levels with partial reduction in total cholesterol but had no effect on HDL and LDL levels (Figures S2O–R and 3K). Higher LDL levels in nitrite treated iNOS^−/−^ mice correlated with increased LDLR and PCSK9 protein expression in the liver (Figure S2S,T) while LDLR was marginally reduced in iNOS^−/−^ mice (Figure S2S). Results of nitrite supplementation in WT mice were as per established literature, and hence are not shown.

### 3.4. Metabolic Homeostasis in Liver and Adipose Tissue in Chow Fed iNOS^−/−^ Mice after Nitrite Supplementation

**Figure 4:**
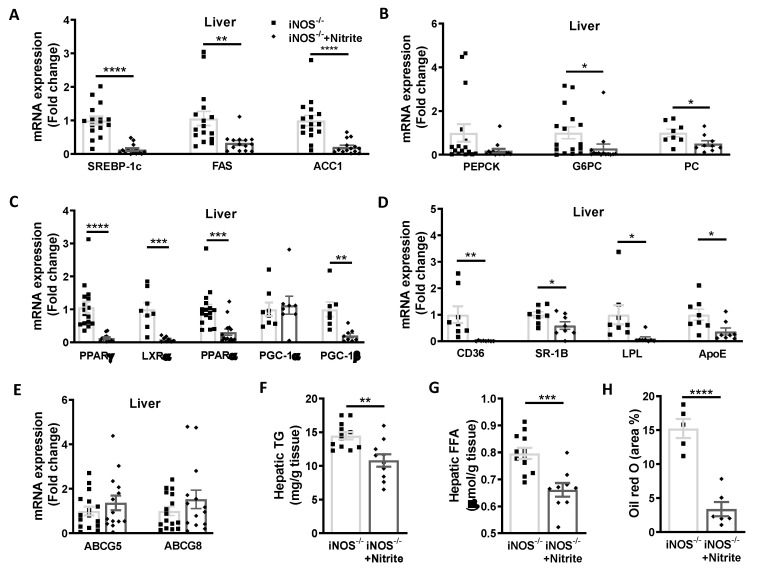
Metabolic homeostasis in liver of chow fed iNOS^−/−^ mice with and without nitrite supplementation. (**A**) qPCR expression of genes involved in lipid synthesis (iNOS^−/−^: *n* = 16, iNOS^−/−^ + Nitrite: *n* = 14): SREBP-1c, FAS, and ACC1. (**B**) qPCR expression of genes involved in gluconeogenesis (iNOS^−/−^: *n* = 16, iNOS^−/−^ + Nitrite: *n* = 14): PEPCK, G6PC, and PC (*n* = 8). (**C**) qPCR expressions of transcriptional regulators involved in lipid synthesis: PPARy (iNOS^−/−^: *n* = 16, iNOS^−/−^ + Nitrite: *n* = 14) and LXRα (*n* = 8) and genes involved in fatty acids oxidation: PPARα (iNOS^−/−^: *n* = 16, iNOS^−/−^ + Nitrite: *n* = 14), PGC-1α, and PGC-1β (*n* = 8). (**D**) qPCR expression of genes involved in lipid uptake (*n* = 8): CD36, SR-B, ApoE, and LPL. (**E**) qPCR expression of genes involved in lipid efflux (iNOS^−/−^: *n* = 16, iNOS^−/−^ + Nitrite: *n* = 14): ABCG5 and ABCG8. Lipid accumulation in liver, (**F**) hepatic triglycerides (iNOS^−/−^: *n* = 12, iNOS^−/−^ + Nitrite: *n* = 10), (**G**) hepatic free fatty acids (iNOS^−/−^: *n* = 12, iNOS^−/−^ + Nitrite: *n* = 10), and (**H**) hepatic Oil red O staining (iNOS^−/−^: *n* = 5, iNOS^−/−^ + Nitrite: *n* = 6) in chow fed iNOS^−/−^ mice with or without nitrite supplementation. Data are represented as mean ± SEM. Black squares: iNOS^−/−^ mice without nitrite supplementation, black diamonds: iNOS^−/−^ mice with nitrite supplementation. * *p* < 0.05, ** *p* < 0.01, *** *p* < 0.001, **** *p* < 0.0001 vs. iNOS^−/−^.

**Figure 5:**
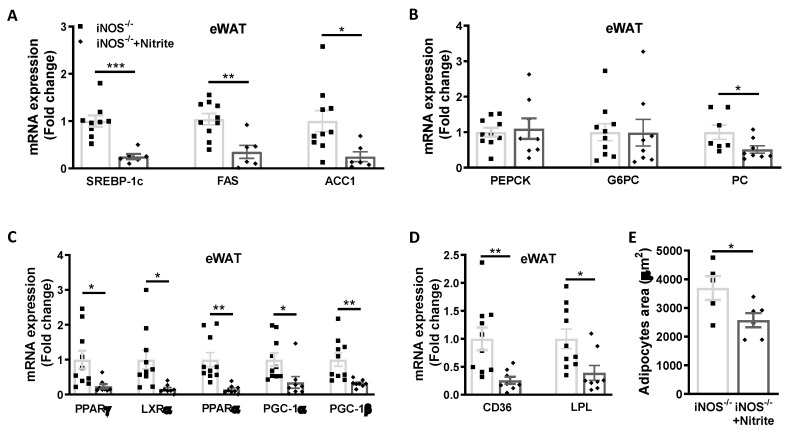
Metabolic homeostasis in adipose tissue of chow fed WT and iNOS^−/−^ mice with and without nitrite supplementation. (**A**) qPCR expression of genes involved in lipid synthesis (iNOS^−/−^: *n* = 10, iNOS^−/−^ + Nitrite: *n* = 6): SREBP-1c, FAS, and ACC1. (**B**) qPCR expression of genes involved in gluconeogenesis (iNOS^−/−^: *n* = 10, iNOS^−/−^ + Nitrite: *n* = 8): PEPCK, G6PC, and PC (iNOS^−/−^: *n* = 7, iNOS^−/−^ + Nitrite: *n* = 8). (**C**) qPCR expressions of transcriptional regulators involved in lipid synthesis (iNOS^−/−^: *n* = 10, iNOS^−/−^ + Nitrite: *n* = 8): PPARy and LXRα and genes involved in fatty acids oxidation: PPARα, PGC-1α, and PGC-1β. (**D**) qPCR expression of genes involved in lipid uptake (iNOS^−/−^: *n* = 10, iNOS^−/−^ + Nitrite: *n* = 8): CD36 and LPL. (**E**) Mean adipocyte area (iNOS^−/−^: *n* = 5, iNOS^−/−^ + Nitrite: *n* = 6) in chow fed iNOS^−/−^ mice with or without nitrite supplementation. Data are represented as mean ± SEM. Black squares: iNOS^−/−^ mice without nitrite supplementation, black diamonds: iNOS^−/−^ mice with nitrite supplementation. * *p* < 0.05, ** *p* < 0.01, *** *p* < 0.001vs. iNOS^−/−^.

Expression of genes involved in the lipid synthesis (SREBP-1c, FAS, and ACC1) was reduced in the liver (Figure 4A) and adipose tissue (Figure 5A) of iNOS^−/−^ mice following nitrite treatment and correlates with the reduction in hepatic lipid accumulation (Figure 4F–H) and marginal decrease (*p* < 0.05) in the adipocytes area in eWAT (Figure 5E). However, genes involved in cholesterol synthesis (HMGCoR, SREBP2) were not altered in the liver and adipose of WT, iNOS^−/−^, and nitrite treated iNOS^−/−^ mice (data not shown). Nitrite treatment in iNOS^−/−^ mice reduced G6PC expression (Figure 4B) with no change in PEPCK expression in the liver (Figure 4B) or eWAT (Figure 5B). Similarly, PC expression was marginally but significantly decreased in liver (Figure 4B) and adipose tissue (Figure 5B) in nitrite supplemented iNOS^−/−^ mice.

Expression of PPARγ and LXRα was normalized after nitrite supplementation in liver (Figure 4C) and adipose tissue (Figure 5C). PPARα and PGC-1β expression in liver (Figure 4C) and adipose tissue (Figure 5C) was significantly reduced following nitrite supplementation to iNOS^−/−^ mice. PGC-1α was decreased in adipose tissue of nitrite treated iNOS^−/−^ mice (Figure 5C) but not in liver (Figure 4C). Expression of CD36, SR-1B, ApoE, and lipolysis gene, LPL in liver (Figure 4D), and CD36 and LPL in adipose tissue (Figure 5D) was regressed by nitrite supplementation in iNOS^−/−^ mice. ABCG5 and ABCG8 expression remained unaltered after nitrite supplementation in iNOS^−/−^ mice (Figure 4E).

### 3.5. Insulin Signaling in Nitrite Supplemented Chow Fed WT and iNOS^−/−^ Mice

**Figure 6:**
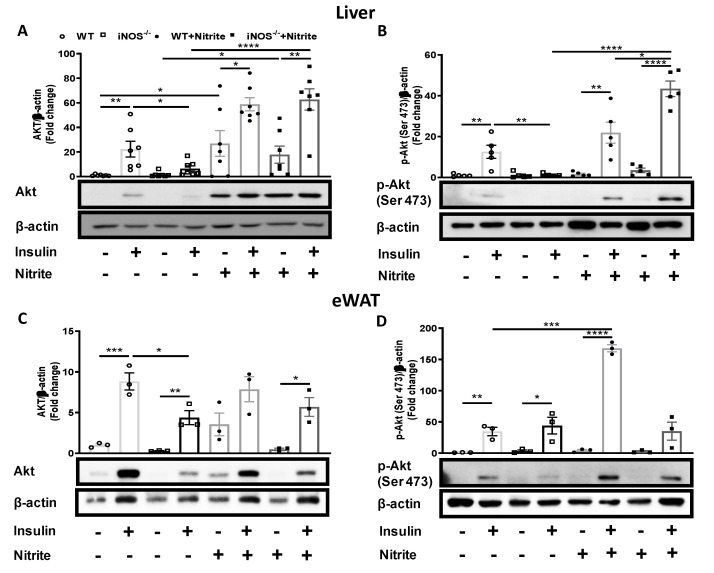
Insulin signaling in chow fed WT and iNOS^−/−^ mice and its alteration by nitrite treatment. Immunoblots of liver (**A**) Akt-1/2/3 (*n* = 7) and (**B**) p-Akt-1/2/3 (*n* = 5). Immunoblots of adipose tissue (*n* = 3) (**C**) Akt-1/2/3 and (**D**) p-Akt-1/2/3. Bar diagrams represent mean ± SEM in chow fed, control, or nitrite treated WT and iNOS^−/−^ mice in both basal and insulin stimulated conditions. White circles: WT; black circles: WT supplemented with nitrite; white squares: iNOS^−/−^; black squares: iNOS^−/−^ supplemented with nitrite. * *p* < 0.05, ** *p* < 0.01, *** *p* < 0.001, **** *p* < 0.0001 between indicated groups.

Metabolic homeostasis is primarily regulated by insulin signaling via Akt in the insulin sensitive organs. Total Akt expression was reduced significantly in the liver and adipose tissue of iNOS^−/−^ mice as compared to WT, and after nitrite treatment was restored in the liver (Figure 6A) but only marginally in the adipose tissue in iNOS^−/−^ mice (Figure 6C). Akt-1/2/3 phosphorylation (Ser473) was also significantly reduced in the liver of iNOS^−/−^ mice as compared to WT, which was completely reversed after nitrite supplementation (Figure 6B). Nitrite significantly enhanced the p-Akt levels in the adipose tissue of WT mice but not in the iNOS^−/−^ mice (Figure 6D).

## 4. Discussion

Single (eNOS) [21], double (eNOS/nNOS) [22], and triple (eNOS, nNOS, and iNOS) [23] NOS knockout mice are IR and display metabolic perturbations. Studies conducted so far on iNOS^−/−^ mice used several dietary fats such as lard or vegetable oils [25,29,32,33,50] among which lard is more obesogenic and diabetogenic [51]. In addition, dietary regimens and protocols to examine the role of iNOS in IR, obesity, and diabetes [25,27,29,33,50] were also different reporting either IR and dyslipidemia [16,31,32,33] or protection against IR as outcome [25,26,27,28,29,30]. The decreased NO bioavailability in endothelial dysfunction, atherosclerosis, diabetes, obesity, and metabolic syndrome is well established. The role of iNOS as a pro-inflammatory agent is also well established but its protective role under normal physiological conditions, and cardiovascular disorders is less investigated. The present comparative study was therefore undertaken in chow fed iNOS KO, and WT mice to systematically assess insulin sensitivity by monitoring systemic (GTT, ITT, PTT), tissue (insulin signaling), biochemical (glucose and lipids), and molecular (lipid and glucose metabolism) parameters, as well as by calorimetry using a comprehensive lab animal monitoring system. Furthermore, metabolic perturbations were evaluated at the hepatic and adipose tissue level by investigating expressions of crucial genes and insulin signaling.

iNOS^−/−^ mice fed on chow diet [30], LFD [32,33], or HFD [25,28,33] exhibited gain in weight and fat mass with no change in food intake [28]. We also observed that weight gain by iNOS^−/−^ mice with similar food intake, correlated both with increase in body fat and decrease in lean mass. In addition, iNOS^−/−^ mice in the present study displayed so far unreported higher BMI and body length (Figure 1C and Figure S1G). On the contrary no change in the body weight in KO mice was reported [27,29,39,50] even with higher chow or HFD intake [25,29]. These observed discrepancies can be ascribed to the differences in the protocols and diets used in the studies [25,27,28,29,30,39].

Non-specific NOS inhibition enhanced serum and hepatic lipids in rodents [35,36] and increased eWAT and perirenal fat deposits [52]. Area of adipocytes was also enhanced in iNOS^−/−^ mice fed on chow diet or HFD diet for 16–18 weeks [25,28,29] suggesting a link between absence of iNOS and increased adiposity. In the present study we observed significant reduction in VCO_2_, heat production, and metabolic rates with no change in the physical activity in chow fed iNOS^−/−^ mice. Low BMR and RMR are associated with metabolic thrift, weight gain, and obesity [53]. Interestingly Nakata et al. reported increase in triglycerides in chow fed iNOS^−/−^, nNOS^−/−^, eNOS^−/−^, *n*/i/eNOS^−/−^ mice [31]. Kakimoto et al. also found increased lipids in iNOS^−/−^ mice 4 weeks after LFD or HFD feeding [33] even though they used C57BL/6N mice which are less prone to obesity due to intact NNT activity. Moreover, Nozaki et al. showed increased circulating and hepatic NEFA in HFD fed iNOS^−/−^ mice while others did not find change in the lipids [26,27,29,30]. Our findings thus confirm the obese phenotype of iNOS^−/−^ mice.

Blood glucose, AUC values of GTT and IIT, as shown in a recently published report [54], were similar to our findings in KO mice. Perreault et al. also showed systemic hyperglycemia [25], and Cha et al. found marginally increased insulin levels [30,39]. Incidentally, in some of the reports, basal glucose levels in WT mice were on the higher side [28,55]. GTT was mostly conducted using 1 g/kg dose of glucose [25,28,29,31,33] while in the present study we used 2 g/kg glucose in a relatively large number (>30) of iNOS^−/−^ mice. Moreover, our finding on PTT support the enhanced gluconeogenesis in KO mice.

Expectedly, total nitrite levels in iNOS^−/−^ mice were significantly less as has also been reported by others [27,28,32,38,39,50] with decreased eNOS and increased nNOS expression in liver and adipose tissue. This might be due to compensatory mechanisms developed due to the loss of a particular NOS gene. Interestingly, low nitrite diet fed mice displayed glucose intolerance, IR, and high circulating lipid levels [19]. Nitrite acts a precursor for NO generation in saliva, stomach, blood, urine, and skin through enzymatic and non-enzymatic mechanisms and it was thus hypothesized that it may compensate the reduced NO availability in the iNOS^−/−^ mice. Nitrite supplementation to iNOS^−/−^ mice reversed insulin sensitivity, insulin levels, augmented lean mass, decreased fat mass and liver weight, with partial yet significant rescue in glucose levels, glucose tolerance, and gluconeogenesis. These findings suggest nutrition based strategies, like use of green leafy vegetables and other nitrite rich foods, against IR. Nitrate/nitrite treatment also improved glucose intolerance in eNOS^−/−^ mice [21,56], reduced fasting blood glucose in db/db mice [42], rescued glucose intolerance and HOMA-IR in diabetic KKA^y^ mice [43], and reversed insulin levels with improvement in GTT and PTT in HFD fed diabetic rats [57]. Reduction in RMR and VO_2_ with no change in RER has also been reported in healthy human volunteers after nitrate supplemented diet [58]. Moreover, long term treatment with nitrate/nitrite also improved blood glucose, insulin sensitivity with decreased insulin and HOMA-IR in WT mice [59] as also observed by us. Likewise, no significant change in LDL levels was observed upon nitrite treatment [19,57]. This can be due to increased PCSK9 expression in liver along with enhanced expression of LDLR. The marginal effect of nitrite on obesity related parameters [19,43,56,57] cannot be explained only on the basis of reversal in NO levels and eNOS expression in KO mice [19]. Partial reversal of adiposity by nitrite supplementation in iNOS^−/−^ mice suggests a role of other regulators in metabolic perturbations. Moreover, recent advances have made us more aware of gut microbiota contribution to metabolic disorders through an axis of communication with adipose tissue regulating body weight and metabolism [60]. However, the role of gut microbiome has not been examined so far in the iNOS^−/−^ mice thus warranting further investigations on these lines.

Gene expression analysis data correlates with the functional and biochemical findings in iNOS^−/−^ mice. Unaltered expression of G6PC and PEPCK in iNOS^−/−^ mice were observed earlier [27], and PPARy expression was also reported to be increased in eWAT of HFD fed iNOS^−/−^ mice [29]. Increase in the expression of SREBP-1c and LPL in the adipose tissue also correlated with profound increase in the circulating NEFA as was observed during iNOS inhibition induced lipolysis in the adipose tissue [61]. PGC-1α induction promotes mitochondrial biogenesis as well as augmented gluconeogenic gene expression and increased lipid oxidation in altered metabolic states [62]. Increased PGC-1α in eWAT of HFD fed iNOS^−/−^ mice was also observed earlier [28]. Lipolysis in the adipose tissue promotes supply of fatty acids and acetyl CoA to the liver to enhance glucose production via PC activation [63]. Induction in PC expression (Figure 2H) supports enhanced gluconeogenesis, which was previously not examined in iNOS^−/−^ mice. Nitrite treatment marginally reversed the induction in PC expression but not that of PGC-1α in the liver (Figure 4C). Increase in PPARy expression in the liver and adipose tissue of iNOS^−/−^ mice was normalized by the nitrite treatment. The present study extensively examined IR and insulin signaling in the liver of iNOS^−/−^ mice and found disrupted insulin signaling unlike earlier studies which show no change [25,27,29]. Interestingly, iNOS^−/−^ mice fed on chow diet showed significant increase in fat mass and marginal reduction in the insulin signaling (PI3K-Akt axis) [30]. Reduction in the sensitivity of insulin signaling in liver and adipose tissue of iNOS^−/−^ mice was rescued by nitrite treatment in liver but not in adipose tissue providing an explanation for the partial recovery of obese phenotype in iNOS^−/−^ mice. The present study, by using a multipronged approach, thus highlights the importance of iNOS in maintaining glucose and lipid homeostasis, and IR.

## 5. Conclusions

The present study was aimed at characterizing adult iNOS^−/−^ mice for IR by systematically evaluating the phenotypic, biochemical, functional parameters and also by limited analysis of important genes. Chow fed adult iNOS^−/−^ mice like other NOS^−/−^ mice exhibited systemic IR, dyslipidemia, and metabolic perturbations. Improvement in IR after nitrite supplementation correlated with compensated NO levels which partially reversed the gluconeogenesis, dysregulated insulin signaling, and weight gain suggesting the beneficial role of homoeostatic iNOS/NO in metabolic regulation. The results obtained thus demonstrate the important contribution of liver and adipose tissue in impacting the insulin sensitivity in iNOS^−/−^ mice.

## Supplementary Materials

The following are available online at https://www.mdpi.com/2076-3921/9/8/736/s1. Figure S1: Systemic metabolic homeostasis and NOS isoforms expression in chow fed WT and iNOS^−/−^ mice. Figure S2: Gross parameters, systemic glucose tolerance, insulin sensitivity, gluconeogenesis and lipids in chow fed iNOS^−/−^ mice with or without nitrite supplementation. Figure S3: Systemic metabolic homeostasis and NOS isoforms expression in chow fed iNOS^−/−^ mice with and without nitrite supplementation. Table S1: List of primary antibodies used and their working dilutions. Table S2: Primers list for qPCR in mice.

## Abbreviations

ABCG5/8	ATP-binding cassette subfamily G member 5/8;
ACC1	Acetyl-CoA carboxylase 1;
ApoE	Apolipoprotein E;
BMI	Body mass index;
BMR	Basal metabolic rate;
CD36	Cluster of differentiation 36;
eNOS	Endothelial-nitric oxide synthase;
eWAT	epididymal white adipose tissue;
FAS	Fatty acid synthase;
FoxO1	Forkhead box O1
G6PC	Glucose-6-phosphatase;
HFD	High fat diet;
HMGCoR	3-hydroxy-3-methyl-glutaryl-coenzyme A reductase;
HOMA-IR	Homeostatic Model Assessment of Insulin Resistance;
iNOS^−/−^	Inducible-nitric oxide synthase knockout;
IPGTT	Intra-peritoneal glucose tolerance test;
IR	Insulin resistance;
ITT	Insulin tolerance test;
KO	Knock out
LDLR	Low-Density Lipoprotein Receptor;
LFD	Low fat diet;
LPL	Lipoprotein Lipase;
LXR	Liver X receptor;
nNOS	Neuronal-nitric oxide synthase;
NO	Nitric oxide;
pAkt	phospho protein kinase B;
PC	Pyruvate carboxylase;
PCSK9	Proprotein convertase subtilisin/kexin type 9;
PEPCK	Phosphoenolpyruvate carboxykinase;
PGC	Peroxisome proliferator-activated receptor-γ coactivator;
PI3K	Phosphoinositide 3-kinases;
PPAR	Peroxisome proliferator-activated receptor;
PTT	Pyruvate tolerance test;
QUICKI	Quantitative insulin-sensitivity check index;
RER	Respiratory Exchange Ratio;
RMR	Resting metabolic rate;
SR-1B	Scavenger receptor, class B type 1;
SREBP-1c	Sterol regulatory element-binding protein 1c;
VLDL	Very-low-density lipoprotein;
WT	Wild type
