# Supplementary material for: Systemic Insulin Resistance and Metabolic Perturbations in Chow Fed Inducible Nitric Oxide Synthase Knockout Male Mice: Partial Reversal by Nitrite Supplementation
Source: Antioxidants (Basel). 2020 Aug 12;9(8):736. doi: 10.3390/antiox9080736 (PMC7465804; doi:10.3390/antiox9080736)
Supplement: Supplementary file 1 [file antioxidants-09-00736-s001.pdf]

Supplementary Tables

Table S1: List of primary antibodies used and their working dilutions

Antibody	Host Species	Source	Cat#	Dilution	Validation
p-Akt(Ser473)	Rabbit	Cell Signaling Technologies	4060	1:2000	Supplier
Akt	Rabbit	Cell Signaling Technologies	4685	1:2000	Supplier
β-actin	Mouse	Genescript	A00730-100	1:10,000	Supplier
eNOS	Rabbit	Santa Cruz Biotechnology	sc-654	1:1000	Supplier
PCSK9	Sheep	R&D systems	AF3888	1:2000	Supplier
LDLR	Rabbit	Biovision	3839-100	1:2000	Supplier
Goat anti rabbit HRP	Goat	Sigma	A0545	1:10,000	Supplier
Anti sheep HRP	Donkey	R&D systems	HAF016	1:1000	Supplier

Table S2: Primers list for qPCR in mice

Gene	Forward Primer Sequence (5'-3')	Reverse Primer Sequence (5'-3')
eNOS	CAACGCTACCACGAGGACATT	CTCCTGCAAAGAAAAGCTCTGG
nNOS	CCAACCCAACGTCATTTCTG	CATAGCTGAGGTCTACCAGG
iNOS	ACCTTGTTCAGCTACGCCTT	CATTCCCAAATGTGCTTGTC
PEPCK	TCTCTGATCCAGACCTTCCAA	GAAGTCCAGACCGTTATGCAG
G6PC	AAGCCAACGTATGGATTCCG	ACAGCAATGCCTGACAAGACT
PC	GGGATGCCCACCAGTCACT	CATAGGGCGCAATCTTTTTGA
SREBP-1c	GGAGCCATGGATTGCACATT	CCTGTCTCACCCCCAGCATA
FAS	GGCATCATTGGGCACTCCTT	GCTGCAAGCACAGCCTCTCT
ACC1	GGACAGACTGATCGCAGAGAAAG	TGGAGAGCCCCACACACA
PPARγ	AGTGGAGACCGCCCAGG	GCAGCAGGTTGTCTTGGATGT
LXRα	GCTCTGCTCATTGCCATCAG	TGTTGCAGCCTCTCTACTTGGA
PPARα	GTCCTCAGTGCTTCCAGAGG	GGTCACCTACGAGTGGCATT
PGC-1α	AACCACACCCACAGGATCAGA	TCTTCGCTTTATTGCTCCATGA
PGC-1β	CGCTCCAGGAGACTGAATCCAG	CTTGACTACTGTCTGTGAGGC
CD36	GCAAAACGACTGCAGGTCAAC	TGGTCCCAGTCTCATTTAGCCA
SR-1B	GGCTGCTGTTTGCTGCG	GCTGCTTGATGAGGGAGGG
ABCG5	TGGATCCAACACCTCTATGCTAAA	GGCAGGTTTTCTCGATGAACTG
ABCG8	TGCCCACCTTCCACATGTC	ATGAAGCCGGCAGTAAGGTAGA
ApoE	AACCGCTTCTGGGATTACCT	CAGTGCCGTCAGTTCTTGTG
LPL	AAGGTCAGAGCCAAGAGAAGCA	CCAGAAAAGTGAATCTTGACTTGGT
18S rRNA	GCAATTATTCCCCATGAACG	GGCCTCACTAAACCATCCAA

* Primers were from Integrated DNA Technology

Supplementary figures

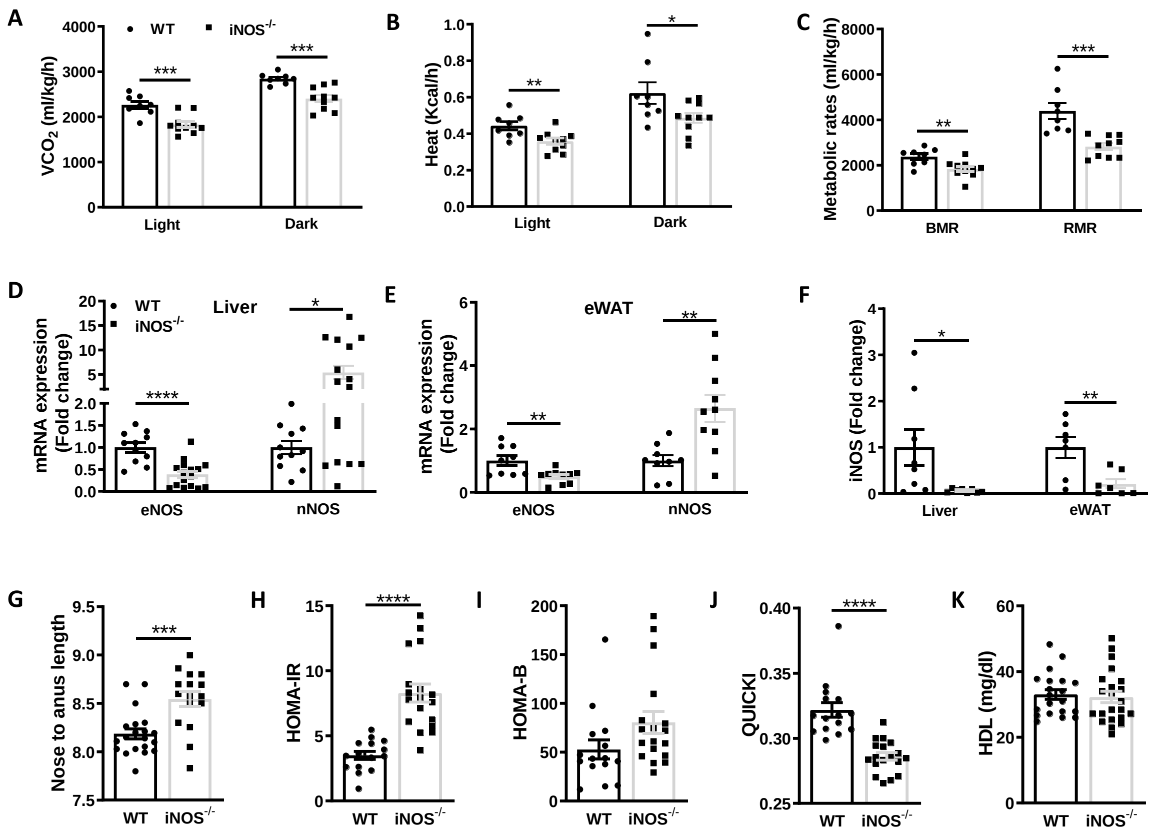

Figure S1: Systemic metabolic homeostasis and NOS isoforms expression in chow fed WT and iNOS$^{-/-}$ mice. Metabolic homeostasis and energy utilisation (WT: *n* = 8, iNOS$^{-/-}$: *n* = 10) (**A**) VCO_2, (**B**) Heat production and (**C**) Metabolic rates (RMR and BMR). NOS isoforms expression (**D**) eNOS and nNOS qPCR gene expression in liver (WT: *n* = 11, iNOS$^{-/-}$: *n* = 16), (**E**) eNOS and nNOS qPCR gene expression in adipose tissue (WT: *n* = 9, iNOS$^{-/-}$: *n* = 10) (**F**) iNOS qPCR gene expression in liver (*n* = 8) and adipose tissue (*n* = 7). Gross measurement of body length (**G)** Nose to anus length (WT: *n* = 20, iNOS$^{-/-}$: *n* = 16). Indexes of insulin sensitivity and β-cell functionality (WT: *n* = 16, iNOS$^{-/-}$: *n* = 18) (**H**) HOMA-IR, (**I**) HOMA-B (**J**) QUCIKI and serum lipids (WT: *n* = 16, iNOS$^{-/-}$: *n* = 22) (**K**) HDL in chow fed WT and iNOS$^{-/-}$ mice. Data

are represented as mean ± SEM. Black circles: WT, Black squares: iNOS$^{-/-}$ mice. * $p <$ 0.05, ** $p < 0.01$, *** $p < 0.001$, **** $p < 0.0001$ vs WT.

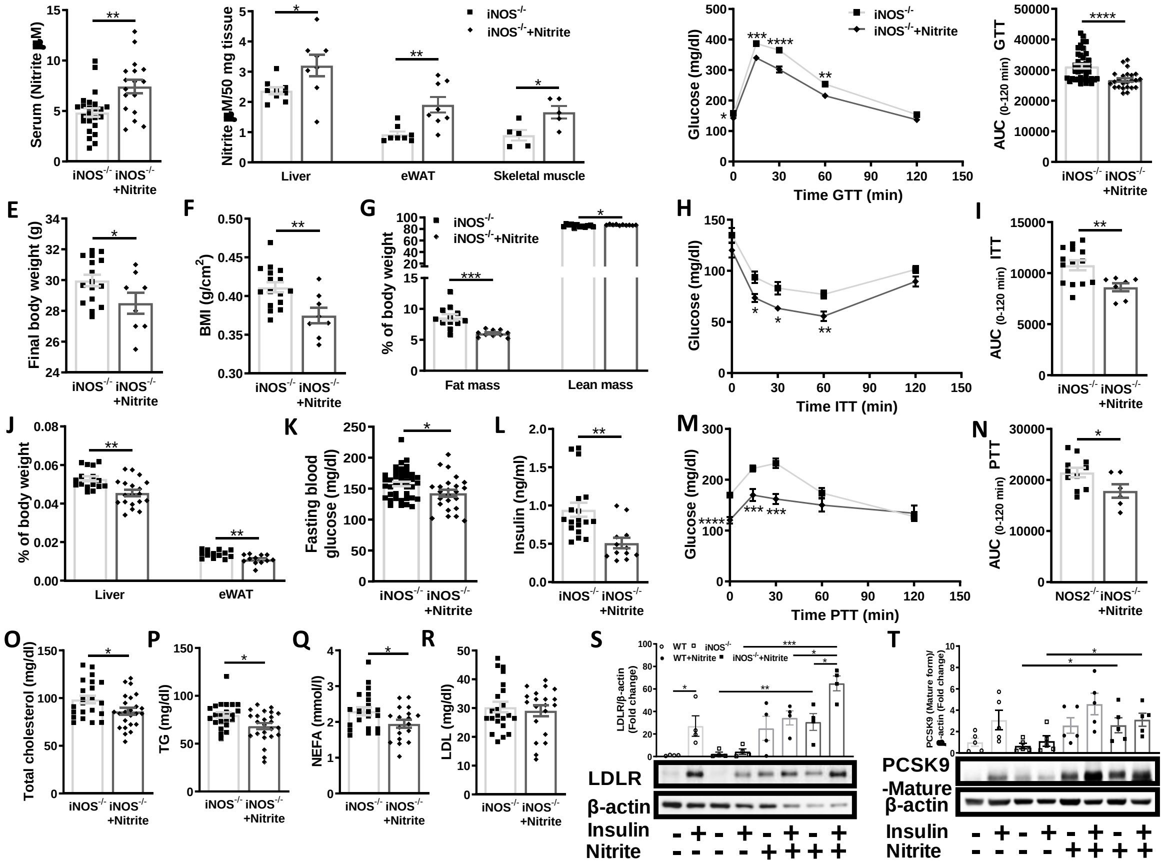

Figure S2: Gross parameters, systemic glucose tolerance, insulin sensitivity, gluconeogenesis and lipids in chow fed iNOS$^{-/-}$ mice with or without nitrite supplementation. (**A**) Total nitrite levels in serum (iNOS$^{-/-}$: n = 24, iNOS$^{-/-}$ + Nitrite: n = 18), (**B**) Total nitrite levels in insulin sensitive tissues—liver (n = 9), white adipose tissue (n = 8) and skeletal muscle (n = 5). (**C**) Intraperitoneal glucose tolerance test (GTT) and (**D**) Area under the curve (AUC) calculated from IPGTT data (iNOS$^{-/-}$: n = 40, iNOS$^{-/-}$ + Nitrite: n = 24). (**E**) Final body weight (iNOS$^{-/-}$: n = 16, iNOS$^{-/-}$ + Nitrite: n = 8), (**F**) Body mass index (BMI) (iNOS$^{-/-}$: n = 16, iNOS$^{-/-}$ + Nitrite: n = 8), (**G**) Whole

body fat mass and lean mass (%) (iNOS$^{-/-}$: *n* = 12, iNOS$^{-/-}$ + Nitrite: *n* = 10), **(H)** Intraperitoneal insulin tolerance test (ITT) and (**I**) AUC calculated from ITT (iNOS$^{-/-}$: *n* = 10, iNOS$^{-/-}$ + Nitrite: *n* = 6), (**J**) Relative liver weight (iNOS$^{-/-}$: *n* = 16, iNOS$^{-/-}$ + Nitrite: *n* =18) and epididymal white adipose tissue weight (eWAT) (*n* = 12), (**K**) Fasting blood glucose levels (iNOS$^{-/-}$: *n* = 40, iNOS$^{-/-}$ + Nitrite: *n* = 24), (**L**) Fasting serum insulin levels (iNOS$^{-/-}$: *n* = 18, iNOS$^{-/-}$ + Nitrite: *n* = 12), (**M**) Intraperitoneal pyruvate tolerance test (PTT) and (N) AUC calculated from PTT (iNOS$^{-/-}$: *n* = 10, iNOS$^{-/-}$ + Nitrite: *n* = 6). Lipid levels in 6 hours fasting serum (iNOS$^{-/-}$: *n* = 22, iNOS$^{-/-}$ + Nitrite: *n* = 18) (**O**) Total cholesterol (TC), (**P**) Triglycerides (TG), (**Q**) Non-esterified free fatty acids (NEFA), (**R**) Low density lipoprotein (LDL). Data are represented as mean ± SEM. Black squares: iNOS$^{-/-}$ mice without nitrite supplementation, Black diamonds: iNOS$^{-/-}$ mice with nitrite supplementation. **p* < 0.05, ***p* < 0.01, ****p* < 0.001, *****p* < 0.0001 vs iNOS$^{-/-}$. (**S**) Immunoblot of LDLR in liver (*n* = 4). (**T**) Immunoblot of PCSK9 in liver (*n* = 5) in chow fed iNOS$^{-/-}$ mice with or without nitrite supplementation. White circles: WT; black circles: WT supplemented with nitrite; White squares: iNOS$^{-/-}$; Black squares: iNOS$^{-/-}$ supplemented with nitrite. **p* < 0.05, ***p* < 0.01, ****p* < 0.001, between indicated groups.

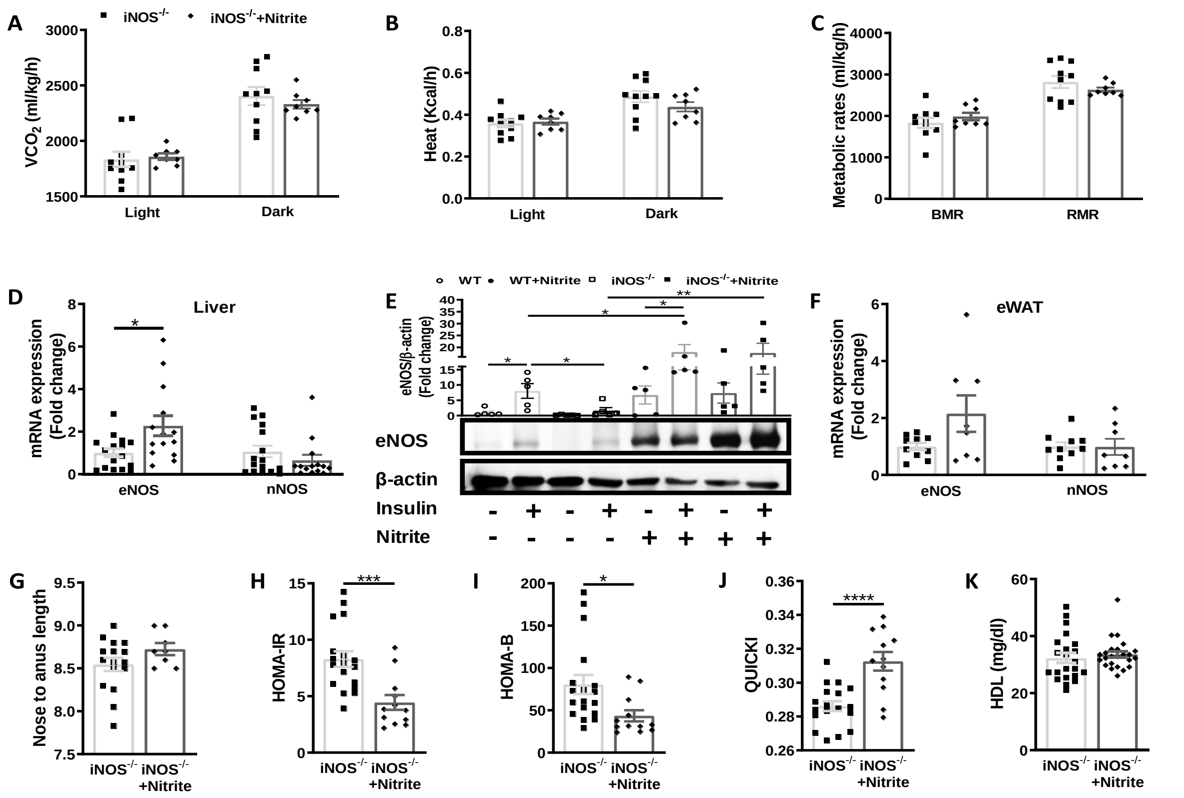

Figure S3: Systemic metabolic homeostasis and NOS isoforms expression in chow fed iNOS$^{-/-}$ mice with and without nitrite supplementation. Metabolic homeostasis and energy utilisation (iNOS$^{-/-}$: n = 10, iNOS$^{-/-}$ + Nitrite: n = 8) (**A**) VCO_2, (**B**) Heat production and (**C**) Metabolic rates (RMR and BMR). NOS isoforms expression (**D**) eNOS and nNOS qPCR gene expression in liver (iNOS$^{-/-}$: n = 16, iNOS$^{-/-}$ + Nitrite: n = 14), (**E**) Immunoblot of eNOS in liver (n = 5), White circles: WT; black circles: WT supplemented with nitrite; White squares: iNOS$^{-/-}$; Black squares: iNOS$^{-/-}$ supplemented with nitrite. *$p < 0.05$, **$p < 0.01$ between indicated groups. (**F)** eNOS and nNOS qPCR gene expression in adipose tissue (iNOS$^{-/-}$: n = 10, iNOS$^{-/-}$ + Nitrite: n = 8). Gross measurement of body length (**G**) Nose to anus length (iNOS$^{-/-}$: n = 16, iNOS$^{-/-}$ + Nitrite: n = 8). Indexes of insulin sensitivity and β-cell functionality (iNOS$^{-/-}$:

$n = 18$, iNOS$^{-/-}$ + Nitrite: $n = 12$) (**H**) HOMA-IR, (**I**) HOMA-B (**J**) QUCIKI and serum lipids (iNOS$^{-/-}$: $n = 22$, iNOS$^{-/-}$ + Nitrite: $n = 18$) (**K**) HDL in chow fed iNOS$^{-/-}$ mice with or without nitrite supplementation. Data are represented as mean ± SEM. Black squares: iNOS$^{-/-}$ mice without nitrite supplementation, Black diamonds: iNOS$^{-/-}$ mice with nitrite supplementation. *$p < 0.05$, ***$p < 0.001$, ****$p < 0.0001$ vs iNOS$^{-/-}$.
